# Impact of Telemedicine use on clinical care indicators of pediatric intensive care units: protocol for a cluster randomized clinical trial

**DOI:** 10.5935/2965-2774.20230223-en

**Published:** 2023

**Authors:** Mariana Motta Dias da Silva, Emanuele König Klever, Jacqueline Castro da Rocha, Gabriela de Oliveira Laguna Silva, Jerusa da Rocha de Amorim, Andressa Dutra Dode, Bárbara Marina Simionato, Luciane Gomes da Cunha, Ana Paula Berni Zaupa, João Ronaldo Mafalda Krauzer, Aristóteles de Almeida Pires, Felipe Cezar Cabral, Tais de Campos Moreira, Hilda Maria Rodrigues Moleda Constant

**Affiliations:** 1 Research Institute, Hospital Moinhos de Vento - Porto Alegre (RS), Brazil; 2 Social Responsibility, Hospital Moinhos de Vento - Porto Alegre (RS), Brazil; 3 Department of Pediatrics, Hospital Moinhos de Vento - Porto Alegre (RS), Brazil; 4 Digital Health, Hospital Moinhos de Vento - Porto Alegre (RS), Brazil

**Keywords:** Randomized controlled trial, Clinical trial protocol, Intensive care, Telemedicine, Intensive care units, pediatric

## Abstract

The objective of this study is to present the protocol of a *cluster* randomized clinical trial to be conducted through the TeleICU project - Qualification of Intensive Care by Telemedicine. The study will consist of a cluster randomized clinical trial, open label, in pediatric intensive care units, with an allocation ratio of 1:1, to compare the intervention group (support of Telemedicine for patients admitted to the pediatric intensive care unit) with a control group (pediatric intensive care unit usual care). The study proposed to select 16 pediatric intensive care units, including 100 participants per site, with a total of 1,600 participants. The intervention group will receive telerounds from Monday to Friday and will have specialists and continuing education activities available. The primary outcome measure will be the length of stay in the pediatric intensive care unit, defined as the difference between the date of discharge of the participant and the date of admission to the intensive care unit. The secondary outcomes will be mortality rate, invasive mechanical ventilation-free days, days using antibiotics, days using vasoactive drugs and days using sedoanalgesia. This study will be conducted in accordance with Resolution 466/12 of the National Health Council, with approval by the Research Ethics Committee of the institutions involved. The present study has the potential to reproduce studies on Telemedicine in intensive care and may make important contributions to care in intensive care units in Brazil and other settings. If Telemedicine shows positive clinical care results compared to conventional treatment, more pediatric patients may benefit.

**ClinicalTrials.gov registry:** NCT05260710

## INTRODUCTION

### Context and rationale

Intensive care units (ICUs) are critical hospital areas marked by great complexity in health care^(1)^ due to the provision of care to patients with more severe clinical conditions.^(2)^ The attention to these patients demands the appropriation of several specific domains of knowledge.^(3)^ From this perspective, the specialization and qualification of human resources is essential for improving the performance of ICUs, both in terms of quality of care and management.^(4)^ Studies have shown better care results in ICUs that have intensivist professionals with specific education and training.^(5,6)^

Despite the positive impact of specialized care, there is a shortage of specialists in Brazilian ICUs.^(7,8)^ According to the *Instituto Brasileiro de Geografia e Estatística* (IBGE), there is inequity in the distribution of intensivist professionals in Brazil,^(9)^ since it is a country with a large demographic area and unequal distribution of resources among regions,^(10)^ especially with regard to the public and private health systems.^(11)^ The shortage of these professionals is still a reality in many Brazilian centers, even with the existence of specific requirements for care in ICUs.^(12)^

Given this scenario, Telemedicine has been identified as a promising tool in the qualification of health care.^(13,14)^ In the context of ICUs, Telemedicine has also been identified as a tool that reduces the length of stay,^(15,16)^ decreases mortality in the units,^(15-17)^ reduces costs,^(15)^ increases satisfaction with care,^(18)^ and is complementary to bedside professionals.^(19)^ Furthermore, its use has the potential to compensate for the lack of intensivists in ICUs because it supports generalist teams.^(20)^

Although there are studies that indicate the effectiveness of Telemedicine in ICUs in many countries,^(21^.^22)^ its impact is not yet consolidated in the literature,^(18,23,24)^ especially in regard to pediatric ICUs. In Brazil, there is a need for more robust studies with well-designed methodologies that demonstrate the results of the use of Telemedicine in ICUs compared to conventional care to build solid evidence of the use of this technology.

Within the Brazilian Unified Health System (SUS - *Sistema Único de Saúde*), there are several opportunities for the development and implementation of Telemedicine. Thus, through the Support Program for Institutional Development of the Unified Health System (PROADI-SUS - *Programa de Apoio ao Desenvolvimento Institucional do Sistema Único de Saúde*), the “TeleICU project - Qualification of Intensive Care by Telemedicine” was developed. In general, the project uses Telemedicine in the form of multiprofessional telerounds for distance education (DE) and discussion of clinical cases in remote ICUs, aiming to systematize care, qualify care, reduce risks for hospitalized patients and improve care indicators of the partner ICUs.^(25)^

### Objectives

To present the protocol of a *cluster* randomized clinical trial (RCT) that will be conducted through the TeleICU project. This RCT aims to evaluate the length of hospital stay in pediatric ICUs, with the secondary objectives of assessing the mortality rate, invasive mechanical ventilation (IMV)-free days and duration of use of vasoactive drugs, broad-spectrum antibiotics and sedation/analgesia.

## METHODS

### Study design

The present protocol refers to a study designed as an RCT, by cluster, open label, in pediatric ICUs, with an allocation ratio of 1:1, in which the hospitals are the clusters, to perform the comparison of the group intervention (support of Telemedicine for patients admitted to the pediatric ICU) with a control group (usual care in the pediatric ICU).

The RCT was registered in the Brazilian Registry of Clinical Trials (ReBEC) on March 22, 2022, and the present protocol provides additional details on the study design and methodology, based on the CONSORT 2010 Statement.^(26-28)^

### Intervention (Telemedicine)

**Figure 1:**
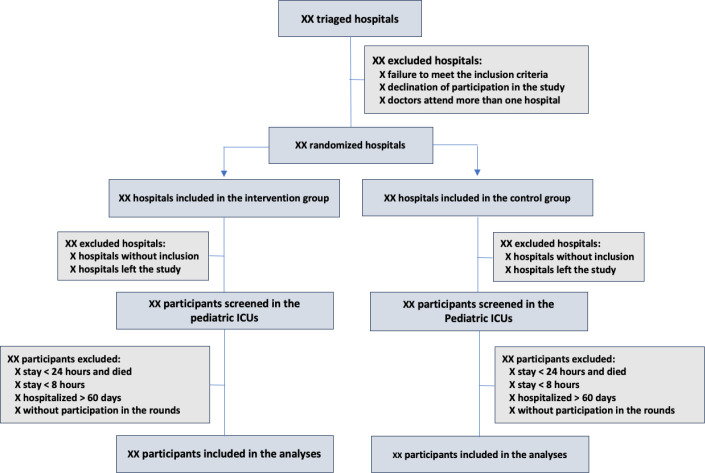
Recruitment of study units and participants.

The intervention group, during the data collection period, will receive telerounds from Monday to Friday and will have specialists available on demand (as cases and specific needs arise, the pediatric ICUs may request evaluation by specialists from the proposing institution) and continuing education activities. The telerounds will be case discussions with pediatric intensivists at a distance and doctors of other specialties on demand for the debate on conduct based on the best scientific evidence. The intensivists of the project will connect through a telemedicine CART (Figure 1S - Supplementary Material) with the pediatric ICU team of the participating centers daily, from Monday to Friday, to perform medical consultations at the bedside to establish diagnoses, guide the therapeutic conduct and perform clinical follow-up.

The intensivist of the project will record the appointments on a digital platform, especially designed for the activity. A treatment plan will be suggested by the project team (clinical management recommendations). The physician of the participating center will be free to decide whether to accept the recommendations. These recommendations will be reassessed 24 hours later in the next teleround. The proposal is to maintain horizontal care, in which the patient is monitored from the first day of hospitalization until discharge.

Concurrently with the period of application of the intervention, continuing education activities will be made available to all professionals of the participating centers’ teams. Education activities will be held monthly and will consist of video classes, discussions of complex cases and DE courses. The video classes will be made available synchronously and asynchronously, with topics chosen according to the needs of each center. The discussions of complex cases will be meetings in which each participating center will be responsible for choosing a case considered a challenge to the team, and medical specialists will be invited to participate in the discussions. The DE courses will be developed by the proposing institution and will be available on a platform specific to the study, with specific themes to qualify care in pediatric intensive care. These courses may be attended at any time by staff of the participating centers.

### Control

The control group will initially maintain the usual face-to-face management offered in the pediatric ICUs at the participating centers and will perform data collection. After completing the collection stage, the intervention will be offered along the same lines as the intervention group. The aim is to provide opportunities for the provision of Telemedicine and to draw on the expertise of the professionals of the proposing institution to enhance the care in all the participating centers.

### Selection of pediatric intensive care units and randomization

For the selection of hospitals, the following methodology was used: generate a list of all Brazilian public hospitals with 100% dedication to SUS care and a pediatric ICU; classify the pediatric ICUs according to their complexity, the project selected the type II pediatric ICUs;^(29)^ categorize the pediatric ICUs according to the number of beds, the pediatric ICUs with 4 to 20 beds moved to the next stage; contact all eligible pediatric ICUs (considering steps 1, 2 and 3) by telephone or e-mail, inviting the person in charge to engage in the project; send a questionnaire to assess feasibility to all those responsible for the pediatric ICUs who expressed interest in participating in the project; considering the answers to the feasibility questionnaire and schedule a meeting with the eligible pediatric ICUs together with the team (pediatric intensivist, nurse, researcher and information technology professional) of Hospital Moinhos de Vento (HMV), so that each specialist can be scored according to preestablished criteria; prepare a ranking with the evaluations of the hospitals and select the 16 centers with the highest scores to participate in the project.^(30)^ It is noteworthy that pediatric ICUs (PICUs) that do not have approval from the Research Ethics Committees involved will be excluded.

Subsequently, the selected PICUs will be randomly allocated to the intervention group or control group at a 1:1 ratio, with blocks of varying sizes (two to four), with dichotomous stratification by the estimated median length of stay in 2020 and 2021 (> 11 *versus* ⋦ 11 days).^(31)^ The generation of the randomization sequence for each stratum will be the responsibility of the statistician of the proposing institution, using R software, ensuring the blinding of the allocation sequence of researchers from the proposing institution.

### Sample size

A total of 16 pediatric ICUs, including 100 participants per location, for a total of 1,600 participants will be selected to detect a decrease of 2.3 days of hospitalization in the intervention units compared to the control unit. To consider the standard deviation (SD), the data from the TeleICU project indicators were used for a SD of 6.2 days in the control units and 7.1 in the intervention group, in addition to an intraclass correlation coefficient of 0.05, power of 80% and alpha of 5%. WinPepi software, version 11.65, was chosen to determine the sample size calculation.

### Eligibility of participants

The eligibility criteria for the participants are patients admitted to the selected pediatric ICUs ≥ 29 days and < 18 years of age, with a stay of more than 24 hours and, in case of death, with a stay of more than 8 hours.

The exclusion criteria are patients with incomplete medical records or missing data, with a stay > 90 days in the ICU, who are in the intervention cluster and did not receive telemedicine care, and those whose guardians did not agree to participate in the study and/or did not sign the informed consent.

The recruitment of study units and study participants will follow the criteria of the 2010 statement of the Consolidated Standards of Reporting Trials (CONSORT) for cluster randomized trials and is detailed in figure 1.

ICU - intensive care unit.

### Data collection

#### Software for data collection

The data will be collected and managed using Research Electronic Data Capture (REDCap®), an electronic tool for capturing, storing and managing data.^(32,33)^ The variables of interest that will be collected are sociodemographic data, such as date of birth, sex, self-reported race and age (calculated from the date of birth) and clinical data, described in chart 1S (Supplementary Material).

#### Training for data collection

Both the intervention and control groups will receive training, in which theoretical and practical contents for the collection and management of clinical data will be addressed through REDCap®. In addition, for the intervention group, training will be offered to the health care team that will include content regarding the use of the digital platform to record the attendance and protocol for the telerounds.

#### Pilot data collection

After the training, all centers will conduct pilot data collection for 2 weeks. This time was based on the previous experience of the TeleICU project with other pediatric ICUs. The objective is to accompany the collector until he or she reaches minimum quality conditions and to provide time for efficiency in data collection to be achieved. This is a form of training and adaptation for the application of data collection forms. During this time, the weaknesses of each collector will be identified, and adjustments will be made according to the specific need of each collector. The centers participating in the control group will start the official collection after 2 weeks, while the centers participating in the intervention group will have another 2 weeks of pilot training with the addition of telerounds in their routine. After this period, the centers will begin the official data collection. All data from the pilot phase will be excluded from the database and will be stored based on the rules of the General Data Protection Law (LGPD - *Lei Geral de Proteção de Dados*).^(34)^

### Monitoring of data collection

#### Monitoring committees

To ensure the scientific integrity of the study, the protection of participants and the credibility of the data, in addition to avoiding conflicts of interest, a steering committee will be formed. The constitution of this committee will provide overall oversight for the project to ensure that it is conducted in accordance with the rigorous standards established in the governance framework for research for health and social care of the department of health and the Guide to Good Clinical Practice.^(35)^

#### Data collection monitoring team

**Figure 2:**
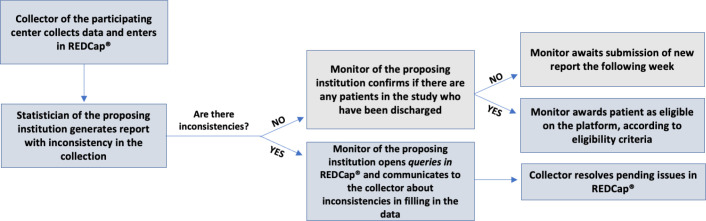
Flowchart of monitoring of data collection.

Data collection will be monitored by a researcher from the project team (monitor) and a statistician. The team will be composed of three research monitors, each of whom will be responsible for certain participating centers and will have direct communication with the collectors of these centers. The monitors will be responsible for reviewing the data weekly, assessing whether the inclusion criteria were met, validating the consistency of the data and verifying that all forms were filled out correctly. For this to be possible, the statistician of the proposing institution will be responsible for reviewing the data weekly, sending reports with the inconsistencies found and necessary adjustments in the collections to the researchers. Figure 2 proposes a detailed flowchart of this process.

REDCap® - Research Electronic Data Capture.

Based on this report, the researchers will open queries in REDCap® (spaces for requesting the review of information), indicating to the collectors of the participating centers the inconsistencies found and what needs to be changed. Once inconsistencies remain, the monitors will inform the collectors by e-mail, message, telephone calls or videoconference so that the doubts are clarified and the inconsistencies resolved. There will also be a follow-up by the monitors regarding the monitoring of the adherence of the pediatric ICUs in the telerounds for the intervention group. In addition, these professionals will evaluate the telerounds to identify possible deviations from the round protocol, as shown in figure 2S (Supplementary Material).

#### Treatment of missing data

We assume that there will be missing data in the research variables. However, monitoring will be performed regularly to check for missing data and inconsistencies, and based on the evidence, the monitors will contact the collectors to make the necessary adjustments.

Data analyses will be performed with the information that is available after the monitoring. Thus, the primary and secondary outcomes will not have data imputation.

### Outcomes

The primary outcome measure will be the length of stay of the patients in the PICU, defined as the difference between the date of discharge and the date of admission to the ICU. The secondary outcomes will be mortality rate, IMV-free days, days using antibiotics, days using vasoactive drugs and days using sedoanalgesia.

### Statistical analysis

Statistical analyses will be performed both by intention-to-treat and by protocol, considering that the study design is a cluster RCT, and data from the observed pediatric ICUs will be analyzed.

The description of the information regarding the PICUs and the characteristics of the participants will be presented for the control and intervention groups, in which the categorical variables will be presented by absolute and relative frequency, while the continuous variables will be first evaluated for normality by means of visual verification of histogram and Shapiro‒Wilk test and presented as the mean and SD or median and interquartile range (IQR), depending on the data distribution, as shown in table 1S (Supplementary Material).

For the primary outcome of the study, a noninferiority analysis will be performed through modeling, considering that the data are correlated in each ICU, by generalized mixed models adjusted for variables of interest to the researchers. Regarding the secondary outcomes, correlated data models will also be used; however, the probability distribution of each outcome will be taken into account. Table 2S (Supplementary Material) describes the data to be analyzed.

Sensitivity analyses will be conducted only for the primary outcome in the intervention group to verify whether adherence to teleround can impact the length of stay.

We will also analyze some exploratory results regarding the days of IMV use, number of days off for each ventilatory support and days off the use of medications such as antibiotics, sedoanalgesia, antifungal and antiviral, water balance during hospitalization and verification of permanence in hospital intensive care (use of IMV or sedoanalgesia) in participants who spent at least 8 days in the pediatric ICU, as shown in table 3S (Supplementary Material).

All statistical analyses will be performed considering a significance level of 0.05. The software to perform all the analyses will be the R program, version 4.2.1 or a later version.

## ETHICS AND DISSEMINATION

### Ethical approval and consent to participate

This study will be conducted in accordance with resolution 466/12 of the National Health Council^(36)^ and was approved by the Research Ethics Committee (REC) of the proposing institution. Subsequently, the project will be submitted to the RECs of the participating centers.

The parents or guardians of the study participants will be asked to sign an informed consent form. Participants ages 6 through 18 will be required to sign an informed consent form. When a patient is unable to consent to participate in the study due to clinical limitations, he or she will be asked to sign a waiver. In addition, rights to use images will be requested.

### Dissemination

The study is expected to make results widely available by disseminating findings in conference presentations and publishing articles in high-impact journals. Moreover, it is expected that the evidence may serve as a basis for the creation of public policies that allow for more adequate and specialized care in all pediatric ICUs in Brazil, even those with a lack of professionals specializing in intensive care or pediatrics.

In addition, through the dissemination of this protocol, we will seek to encourage the reproducibility of similar studies in realities of access to health care different from those found in Brazil so that the potential of telemedicine can be more widely verified and the results of this intervention can be measured in different socioeconomic, cultural and health contexts.

## DISCUSSION

This article describes the protocol of the TeleICU project, which is a multicenter, clustered, open-label RCT study. The main objective is to evaluate the impact of telemedicine on clinical care indicators.

The present study has the potential to reproduce studies on telemedicine in intensive care and may make important contributions to ICU care in Brazil and in other settings. In addition, the study will introduce telemedicine as a way to train professionals who work in ICUs, even those who are not intensivists, as occurs in many Brazilian units. Thus, the study proposes a model that offers benefits when incorporated into the SUS.

The limitations of this protocol relate to the time difference between the participating centers for the start of activities. This is due to the impossibility of controlling the procedures of the respective ethics committees in the processes. In addition, dependence on internet resources impacts the occurrence of telerounds only and exclusively at the bedside. In this sense, we considered this factor to intervene in the analysis of the study.

## CONCLUSION

This protocol presents an innovative panorama because it not only proposes telemedicine in intensive care but also uses this tool to promote equity in regions where there is a lack of specialized professionals in PICUs and for continuing education in places far from large centers in Brazil. Thus, it represents a research scenario little explored thus far. If telemedicine shows positive clinical care results in relation to conventional treatment, more pediatric patients may benefit from the technology as a form of intensive health care.
